# Development of a method to rapidly assess resistance/susceptibility of Micro-Tom tomatoes to Tomato yellow leaf curl virus via agroinoculation of cotyledons

**DOI:** 10.1186/s13104-021-05651-3

**Published:** 2021-06-23

**Authors:** Tomoaki Mori, Kosuke Takenaka, Fumiya Domoto, Yasuhiro Aoyama, Takashi Sera

**Affiliations:** 1 Department of Synthetic Chemistry and Biological Chemistry, Graduate School of Engineering, Kyoto University, Kyotodaigaku-Katsura, Nishikyo-ku, Kyoto, 615-8510 Japan; 2 Department of Applied Chemistry and Biotechnology, Graduate School of Interdisciplinary Science and Engineering in Health Systems, Okayama University, Tsushima-Naka, Kita-ku, Okayama, 700-8530 Japan

**Keywords:** *Agrobacterium*, Agroinoculation, Cotyledon, Micro-Tom, Tomato yellow leaf curl virus

## Abstract

**Objective:**

Tomato yellow leaf curl virus (TYLCV) is one of the pathogens severely damaging tomato crops. Therefore, methods to treat or prevent TYLCV infection need to be developed. For this purpose, a method to conveniently and quickly assess infection of tomatoes by TYLCV is desired. In the present study, we established a quick method to evaluate TYLCV infection using cotyledons of Micro-Tom, a miniature tomato cultivar.

**Results:**

First, we constructed a binary plasmid harboring 1.5 copies of the TYLCV genome and transformed *Agrobacterium* with the plasmid. By injecting agroinoculum from the resulting transformant into the branches of Micro-Tom, we confirmed the susceptibility of Micro-Tom to TYLCV. To shorten the evaluation process of TYLCV infection further, we agroinoculated cotyledons of Micro-Tom 10 days after sowing seeds. We consistently observed typical symptoms of TYLCV infection on true leaves 10 days after agroinoculation. Molecular analysis detected TYLCV progeny DNA in all leaves demonstrating symptoms 6 days after agroinoculation. Therefore, our new protocol enabled assessment of TYLCV infection within 20 days after sowing seeds. Thus, agroinoculation of Micro-Tom cotyledons will accelerate the process of screening TYLCV-resistant Micro-Toms and enable screening of larger numbers of plants more quickly, contributing to the development of TYLCV-resistant tomatoes.

**Supplementary Information:**

The online version contains supplementary material available at 10.1186/s13104-021-05651-3.

## Introduction

Tomato yellow leaf curl virus (TYLCV, genus *Begomovirus*, family *Geminiviridae*) causes tomato yellow leaf curl disease, one of the most devastating diseases of cultivated tomato, and severe economic losses estimated at billions of dollars each year [1]. TYLCV, which is transmitted by the whitefly *Bemisia tabaci*, is quickly spreading to many countries all over the world [2]. The most popular approach to the control of TYLCV is the breeding of TYLCV-resistant tomatoes. However, all the commercially available hybrids today are tolerant of rather than immune to the virus [3]. Therefore, development or establishment of a more effective approach to TYLCV control is desired.

To this end, development of a method to conveniently and quickly assess resistance/susceptibility of bred or engineered cultivars to TYLCV is desired. Commercially-available tomato cultivates (such as Moneymaker and Momotaro) producing large tomato fruits had been already known to be susceptible to TYLCV by using an agroinoculation method [4, 5]. However, a large space is necessary to grow these large tomato plants (170 to 180 cm tall or taller) and it takes long time to assess the infection by TYLCV due to a long life cycle (120–180 days or longer). Among *Solanum lycopersicum* cultivars, a miniature dwarf tomato, Micro-Tom, is now attractive as a model tomato cultivar due to its small plant size (10–20 cm tall), short life cycle (70–90 days), and easy transformation with foreign genes using *Agrobacterium* [6]. Therefore, Micro-Tom seems to be a model tomato cultivar suitable to evaluate a newly developed approach to TYLCV control quickly in a limited space. Micro-Tom has been also used as a host plant for the study of pathogenesis of fungi, bacteria, and viruses [7, 8]. However, to our best knowledge, there is only one study of infection of Micro-Tom by TYLCV [8]. In the previous study, Micro-Tom was for the first time demonstrated to be susceptible to TYLCV by inoculation with viruliferous whiteflies. Although whitefly inoculation occurs naturally and is a popular protocol for begomovirus infection, agroinoculation has several advantages, such as easier and more convenient maintenance of the inoculum, compared to whitefly inoculation. However, the susceptibility of Micro-Tom to TYLCV introduced by agroinoculation has never been reported. Because Micro-Tom is genetically different from other tomato cultivars [9], it is necessary to experimentally assess the susceptibility to TYLCV.

In the present study, we first investigated whether Micro-Tom was susceptible to TYLCV by agroinoculation. Then, to shorten the whole screening process of TYLCV infection, we explored the feasibility of agroinoculation of cotyledons of Micro-Tom at the cotyledon stages although viral replication and transport depend on the development stage of the inoculated tissues and viral DNA concentration of TYLCV is practically imperceptible in non-dividing tissues such as cotyledons and older true leaves [10, 11]. We demonstrated that Micro-Tom was susceptible to TYLCV by agroinoculation and that Micro-Tom developed typical symptoms of TYLCV infection by agroinoculation of its cotyledons within 20 days after sowing the seeds.

## Main text

### Materials and methods

#### Plant materials

Seeds of the *L. esculentum* cultivar Micro-Tom were purchased from Tomato Growers Supply (Fort Myers, FL, USA). The seedlings were used for agroinoculation when the cotyledons had expanded fully and the true leaves were 3–5 mm long. It generally took 10 days after sowing seeds to reach the growth stage.

#### Agroinoculation of branches or seedlings of Micro-Tom

*Agrobacterium tumefaciens* strain C58C1Rif^R^ (a gift from Dr. Hiroshi Ezura) containing pBI-TYLCV(1.5) (see Additional file 1: Supplementary Method for the construction) was grown at 30 °C in Luria–Bertani (LB) medium containing ampicillin (50 µg/ml) and kanamycin (100 µg/ml) until the optical density at 600 nm was 1.5. After brief centrifugation of 3 ml of the culture, the resulting pellet was resuspended in 1 ml of the inoculation medium [10 mM Tris–HCl (pH 7.0)/10 mM MgCl_2_]. The suspension was injected into the seventh branches of two Micro-Tom plants 4 weeks after seedling. Alternatively, the abaxial side of each cotyledons of young Micro-Tom seedlings described above was scratched with a toothpick, and the suspension was injected into the center of each scratch with a 1-ml syringe. The injection was given to two to four seedlings per experiment and repeated six times. The injected plants were covered with a plastic dome to keep them moist and transferred to a growth chamber (25 °C, 16 h light-8 h dark). The next day, the plastic dome was removed and inoculated plants were grown until symptoms appeared.

## Results

### Generation of agroinoculum harboring partial tandem copies of TYLCV genome

**Fig. 1:**
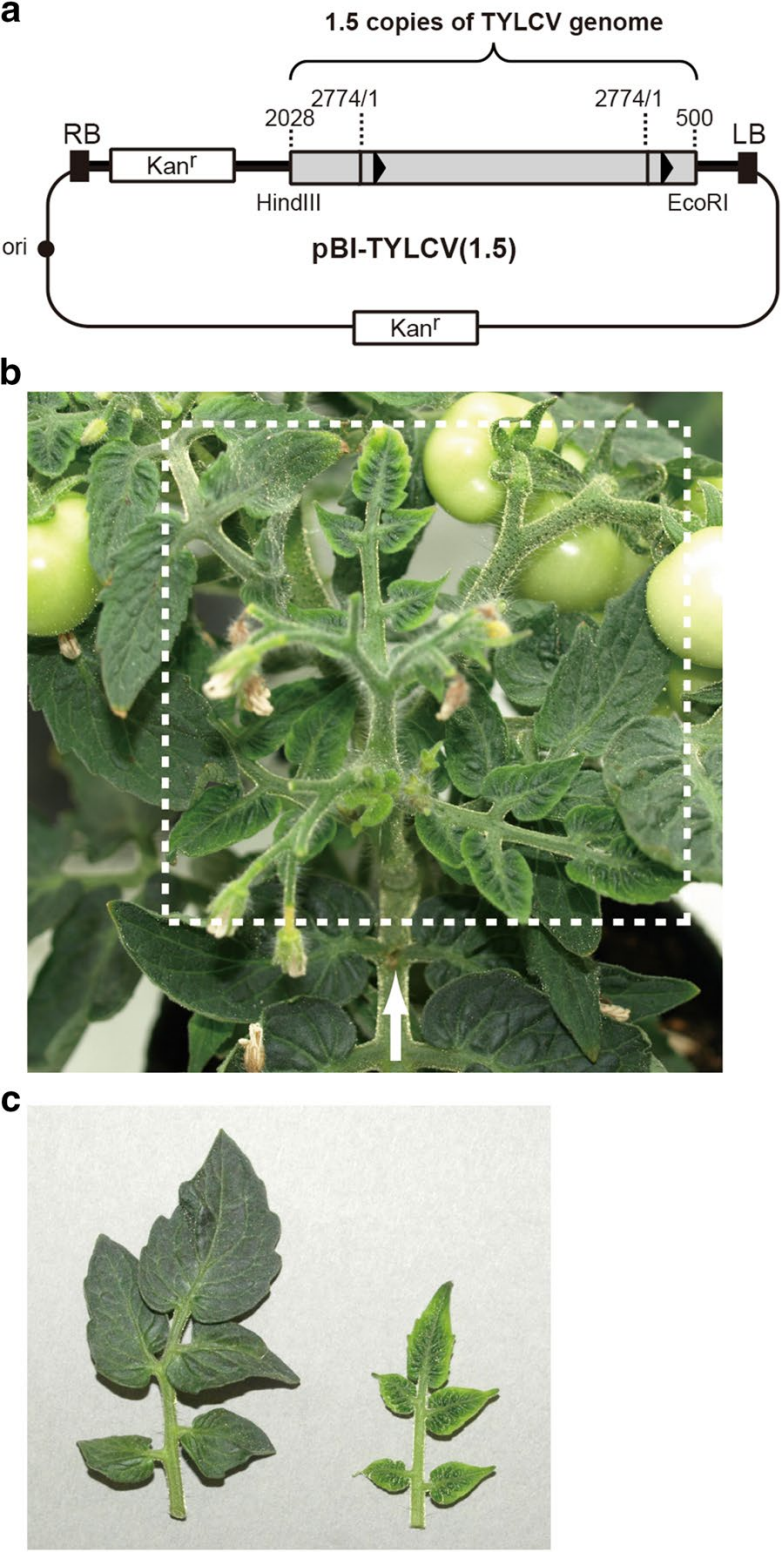
Agroinoculation of Micro-Tom. **a** Schematic diagram of the construct pBI-TYLCV(1.5). The grey box indicates partial tandem copies of the TYLCV genome. The numbers above the boxes indicate their location (in nt) in the TYLCV genome. The two closed triangles indicate stem-loop regions of TYLCV. RB, a T-DNA right border and LB, a T-DNA left border. **b** Axillary bud (shown within a white dotted frame) emerging after injection of agroinoculum harboring partial tandem copies of TYLCV genome into a tenth branch of Micro-tom. Typical symptoms of TYLCV were observed in the bud. The white arrow indicates the location of the injection. **c** Comparison of the leaf (right) from the infected axillary bud with a corresponding leaf (left) from a noninfected healthy control

Among TYLCV strains, we chose the TYLCV-Israel (TYLCV-IL) strain, which was first reported in Israel in 1930 [12], for our study because tomato yellow leaf curl disease due to TYLCV-IL has been spreading around the globe. Among TYLCV strains, the TYLCV-IL strain has the largest number of isolates, and they have been identified in at least 17 countries in diverse areas of the world, including Spain, Tunisia, Egypt, Japan, and the U.S. [12]. For agroinoculation of a geminivirus, a binary vector harboring more than one copy of a geminivirus genome together with two replication origins (or conserved stem-loop regions) is required because a single-stranded TYLCV genomic DNA is generated by replication between two plus-strand origins [13]. Therefore, we introduced 1.5 copies of TYLCV genomic DNA between right and left borders of a binary vector pBI121. The resulting infectious clone pBI-TYLCV(1.5) contains nt 2028–2774, nt 1–2774, and nt 1–500 of the TYLCV genome, where the stem-loop region is nt 14–172 (Fig. 1a). *A. tumefaciens* strain C58C1Rif^R^ was transformed with pBI-TYLCV(1.5) and the resulting transformant was used for agroinoculation.

### Agroinoculation of branches of Micro-Tom

First, we examined whether Micro-Tom was susceptible to TYLCV. Therefore, we grew Micro-Tom 4 weeks after seedling, and then injected the above agroinoculum into the center of the seventh branches. Approximately 40 days after agroinoculation, clear symptoms such as shrunken leaves and laminae curled between the veins were observed as shown in Fig. 1b, c. We observed the same symptoms on Micro-Tom plants by agroinoculation into true leaves as well. Thus, although Micro-Tom is genetically different from other tomatoes, it was experimentally demonstrated for the first time that the cultivate is susceptible to TYLCV by agroinoculation.

### Agroinoculation of cotyledons of Micro-Tom

**Fig. 2:**
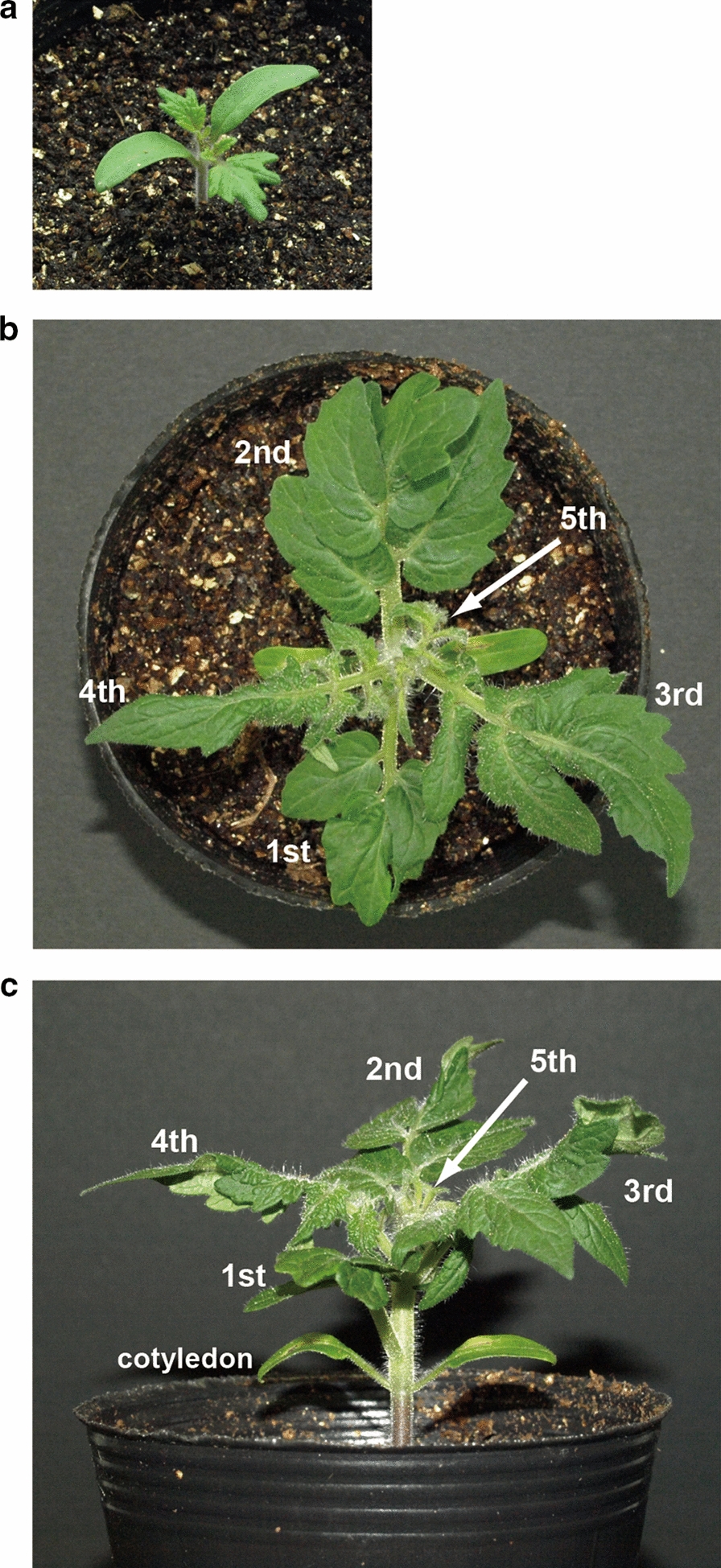
Agroinoculation of Micro-Tom seedlings. **a** A seedling on the 10th day after sowing. Agroinoculum harboring partial tandem copies of TYLCV genome was injected into abaxial sides of cotyledons of such young seedlings. A Micro-Tom seedling 10 days post-inoculation (just before agroinoculation) from above (**b**) and from the side (**c**). 1st, first true leaf; 2nd, second true leaf; 3rd, third true leaf; 4th, forth true leaf; and 5th, fifth true leaf

Although we demonstrated the susceptibility, it takes a long time (e.g., > 2 months) to assess whether (engineered) Micro-Tom plants are resistant to TYLCV by using a conventional agroinoculation method described above. Therefore, to shorten the whole screening process of TYLCV agroinfection, we explored the feasibility of agroinoculation of cotyledons of Micro-Tom. Ten days after sowing Micro-Tom seeds, we scratched the abaxial side of both cotyledons of each young seedling (Fig. 2a) with a toothpick, and injected the above agroinoculum into the center of each scratch with a 1-ml syringe. After agroinoculation, we carefully monitored development of TYLCV symptoms on agroinoculated seedlings every day. Third and fourth true leaves that emerged after agroinoculation started indicating symptoms such as leaf deformation 8 days after agroinoculation. On the 10th day (mean ± standard error; 10.0 ± 0.3 days) after agroinoculation, typical symptoms of TYLCV infection were clearly observed as shown in Fig. 2b, c. The third and fourth true leaves were curled downward and narrowed, and the leaf laminae were wavy between the veins. Severe size reduction in the top (fifth) true leaves was consistently observed. In contrast, first and second true leaves, which had already emerged at agroinoculation, did not show any symptom at all.

### Analysis of TYLCV progeny DNA

**Fig. 3:**
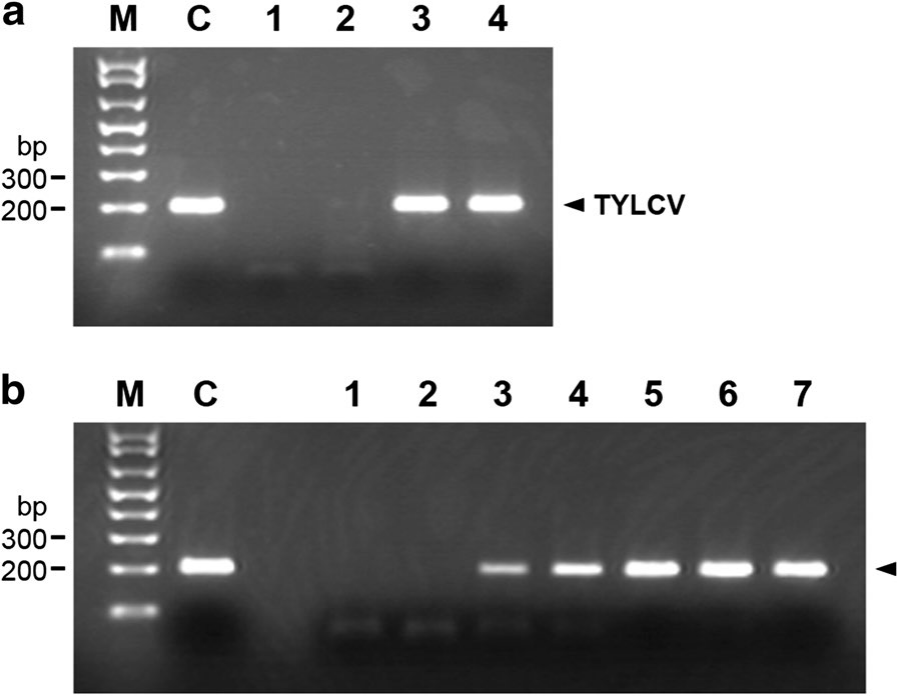
Analysis of DNA extracted from true leaves of Micro-Tom seedlings after inoculation. PCR products amplified from DNA extract with TYLCV-specific primers were analyzed on 2% agarose gel. **a** Analysis of DNA extracted from true leaves of Micro-Tom seedlings 10 days post-inoculation. *Lane M:* a DNA size marker (TrackIt 1 kb Plus DNA Ladder, Invitrogen), *lane C:* PCR product using pBS-TYLCV as a template, *lane 1:* PCR products from the first true leaf, *lane 2:* PCR products from the second true leaf, *lane 3:* PCR products from the third true leaf, *lane 4:* PCR products from the fourth true leaf. **b** Analysis of DNA extracted from third true leaves of Micro-Tom seedlings 4–10 days post-inoculation. *Lane M:* a DNA size marker (TrackIt 1 kb Plus DNA Ladder), *lane C:* PCR product using pBS-TYLCV as a template, *lanes 1*–*7:* PCR products from the third true leaves 4–10 days post-inoculation, respectively. TYLCV viral DNA was detected on the 6th day post-inoculation

TYLCV infection was also confirmed by analysis of TYLCV progeny genome. We first analyzed true leaves of infected seedlings 10 days after agroinoculation. Total DNA was isolated from each true leaf of TYLCV-infected seedlings as described in Additional file 1: Supplementary Method. PCR was then performed using each isolated DNA as a template and a set of TYLCV-specific primers as described in Additional file 1: Supplementary Method. As shown in lanes 3 and 4 of Fig. 3a, TYLCV progeny DNA was detected in both the third and fourth true leaves demonstrating typical symptoms of TYLCV infection. On the other hand, no progeny viral DNA was detected at all in the symptom-free first and second true leaves, which had already emerged at agroinoculation, as shown in lanes 1 and 2 of Fig. 3a. This phenomenon corresponds to previous reports: TYLCV accumulates preferentially in tissues containing dividing cells [14] and the virus spreads following the photoassimilate pathway [11]. First and second true leaves seemed to be too mature for TYLCV to spread to these leaves.

In our experiments, the third true leaves constantly demonstrated symptoms 10 days after agroinoculation. Therefore, we next examined when TYLCV progeny DNA first emerged on the third true leaves. We collected leaf tissue from third true leaves of agroinoculated Micro-Tom seedlings everyday from 4 days through 10 days after agroinoculation and analyzed TYLCV progeny DNA by PCR of DNA isolated from each leaf tissue. As shown in Fig. 3b, no detectable PCR product was observed by the 5th day after agroinoculation and a PCR product corresponding to TYLCV progeny DNA was first detected on the 6th day after agroinoculation. This result demonstrated that our agroinoculation protocol enabled us to assess whether wild-type (or engineered) Micro-Toms were infected by TYLCV within only 16 days after sowing seeds when leaf tissues were analyzed by PCR.

## Discussion

TYLCV is transmitted to tomato plants by the whitefly *Bemisia tabaci* in a persistent and circulative manner [15]. Therefore, inoculation with viruliferous whiteflies is a popular inoculation method. However, agroinoculation has several advantages over the whitefly inoculation. Infection by agroinoculation is stronger than inoculation with whiteflies [16]. While agroinocula like *Escherichia coli* are easily maintained at − 80 °C for long periods, maintenance of living whiteflies is very laborious and poses a potential threat to the environment [17].

A previous study implied that the usefulness of agroinoculation as a virus delivery system in breeding programs for TYLCV resistance is questionable because wild type tomato species that are resistant to TYLCV in field- and whitefly-mediated transmission tests were infected by agroinoculated TYLCV [16]. However, we successfully generated transgenic *Arabidopsis thaliana* plants that were immune to agroinoculated Beet severe curly top virus [18]. By using the same methodology, the generation of transgenic Micro-Tom tomatoes resistant to TYLCV is in progress in this laboratory [19].

To our best knowledge, only one study on infection of Micro-Tom by TYLCV has been reported [8]. In this study, 2-week-old Micro-Tom plants were inoculated with viruliferous whiteflies. Typical symptoms appeared 3 weeks postinoculation. That is, it takes at least 5 weeks after sowing seeds to assess TYLCV infection by using whiteflies in the protocol. In the present study, we for the first time demonstrated susceptibility of Micro-Tom to TYLCV by agroinoculation. Furthermore, we agroinoculated cotyledons of Micro-Tom for the first time. Previous studies demonstrated that TYLCV accumulates preferentially in tissue containing dividing cells [14]: in no-dividing tissues, such as cotyledons and older leaves, viral DNA concentration is practically imperceptible [10, 11]. However, in the present study, we demonstrated that agroinoculation of cotyledons of Micro-Tom resulted in systemic infection of TYLCV. Thus, by agroinoculating cotyledons of Micro-Tom, we shortened the whole process of screening for TYLCV infection after sowing seeds to 20 days by analysis of phenotypes of agroinoculated seedlings and to 16 days by PCR analysis of DNA extracted from leaf tissues. As a result, less space than that for conventional inoculation protocols is necessary for our protocol. Consequently, our agroinoculation protocol enables screening of greater numbers of tomato plants than the currently available protocol for Micro-Tom. We hope that our protocol will contribute to the development of TYLCV-resistant (or immune) tomatoes by accelerating the screening process.

## Limitations

This study was conducted using the TYLCV-IL strain, which was first reported in Israel in 1930. Since then, more than ten TYLCV strains such as TYLCV–Mild and Tomato yellow leaf curl Sardinia virus–Spain have been reported. To confirm the efficacy of our method further, future studies using other TLYCV strains are desired.

## Supplementary Information

**Additional file 1**: Description of data: Plasmid constructions and Detection of TYLCV genomes in infected Micro-Tom.

## Data Availability

All data generated or analyzed during the current study are includes in this published article and its Additional files.

## Abbreviations

TYLCV: Tomato yellow leaf curl virus
TYLCV-IL: TYLCV-Israel
