# Supplementary material for: Development of a method to rapidly assess resistance/susceptibility of Micro-Tom tomatoes to Tomato yellow leaf curl virus via agroinoculation of cotyledons
Source: BMC Res Notes. 2021 Jun 23;14:237. doi: 10.1186/s13104-021-05651-3 (PMC8220776; doi:10.1186/s13104-021-05651-3)
Supplement: Supplementary file 1 — Additional file 1: Description of data: Plasmid constructions and Detection of TYLCV genomes in infected Micro-Tom. [file 13104_2021_5651_MOESM1_ESM.docx]

**Supplementary Method**

***Plasmid constructions***

The whole genome of TYLCV could not be amplified from DNA (a gift from Ryohey Arimoto) isolated from tomatoes infected by the TYLCV-Israel (TYLCV-IL) strain. Therefore, the whole genomic DNA was divided into two parts. Each part was amplified from total DNA using a primer set of 5’-CATCATTACAAGCTTCCTTTGGGCTTAGGTCTAGATGTCCACATAAATA-3’ and 5’-ACCTCTTACCCACTCTGTGAGTAATTCCAGATCCAC-3’ and another primer set of 5’-CTGGAATTACTCACAGAGTGGGTAAGAGGTTCTGTG-3’ and 5’-CCTAAGCCCTGCAGTGTACAGTAATGATGCGTGGTACAACGTCATTGAT-3’, respectively, and digested with DraIII. These two DNA fragments were ligated and cloned into the HindIII/PstI sites of pBluescriptII KS+ (Agilent Technologies, Santa Clara, CA, USA) to generate pBS-TYLCV harboring a whole TYLCV genome.

The plasmid pBI-TYLCV(1.5) used for agroinoculation was generated in two steps. Firstly, 0.5 copy of TYLCV genome was cloned into the binary plasmid pBI121 (Invitrogen). Namely, 0.5 copy of TYLCV genome was amplified from pBS-TYLCV using a primer set of 5’-GGGCCCAAGCTTGAGCTCGGATCCTGTACACCTTTGGGCTTAGGTCTAGATGTCCACATA-3’ and 5’-CCCGGGGAATTCTGGAAATGATTATATCGCCTGGTCGCTTCGACATAG-3’ and then cloned into the HindIII/EcoRI sites of pBI121 to generate pBI-TYLCV(0.5). Secondly, a DNA fragment harboring one copy of TYLCV genome was excised from pBS-TYLCV with HindIII and BsrGI and then cloned into the HindIII/BsrGI sites of pBI-TYLCV(0.5) to construct pBI-TYLCV(1.5).

***Detection of TYLCV genomes in infected Micro-Tom***

DNA was isolated from tomato leaves by using cetyltrimethylammonium bromide (CTAB) [1]. A piece (5 mm × 10 mm) of a fresh leaf from Micro-Tom was ground in 200 μl of extraction buffer (3% CTAB/100 mM Tris-HCl (pH8.0)/1.4 M NaCl/0.2% (v/v) 2-mercaptoethanol/20 mM EDTA) with a pestle and mixed with additional 300 μl of extraction buffer. The mixture was incubated at 60°C for 30 min. Then, 400 μl of chloroform was added to the mixture, shaken well, and centrifuged at room temperature for 5 min. From the aqueous phase, DNA was precipitated with 280 μl of isopropanol. The DNA pellet was resuspended in 100 μl of TE (pH 8.0) and phenol extraction was performed. DNA was precipitated from the aqueous phase with ethanol and resuspended in 50 μl of TE (pH 8.0).

The TYLCV genome fragment was amplified by using total DNA (>0.15 μg) isolated from infected Micro-Tom and a primer set of 5’- ATAATGAGCCCAGTACCGCAACCGTGAAGA-3’ and 5’- GGCGTTTTCAGTATGGTTCTCGTACTTGGC-3’. The PCR sample (0.5 μl) was analyzed on 2% agarose gel. The gel was photographed under UV irradiation.

**References**

[1] Rogers S, Bendich AJ. Extraction of DNA from milligram amounts of fresh, herbarium and mummified plant tissues. Plant Mol Biol. 1985;5:69–76.
